# Factors associated with nursing care rationing in Poland: A cross-sectional observational study

**DOI:** 10.1016/j.ijnsa.2025.100413

**Published:** 2025-08-27

**Authors:** Daria Schneider-Matyka, Kamila Rachubińska, Anna Maria Cybulska, Mariusz Panczyk, Przemysław Ustianowski, Ireneusz Walaszek, Dorota Ćwiek, Elżbieta Grochans, Małgorzata Szkup

**Affiliations:** a Department of Nursing, Pomeranian Medical University in Szczecin, Szczecin, Poland; b Department of Education and Research of Health Sciences, Warsaw University, Warsaw, Poland; c Department of Social Nursing, Pomeranian Medical University in Szczecin, Szczecin, Poland; d Department of Obstetrics and Pathology of Pregnancy, Pomeranian Medical University in Szczecin, Szczecin, Poland

**Keywords:** rationing nursing care, stress, emotional intelligence, nurses

## Abstract

**Background:**

**:** Nursing care rationing is a global issue, but its intensity may vary across countries depending on available resources and healthcare system characteristics.

**Objective:**

**:** This study aimed to examine factors associated with the severity of nursing care rationing among Polish nurses.

**Design:**

**:** Cross-sectional observational study.

**Setting(s):**

**:** Two university clinical hospitals in Szczecin, Poland.

**Participants:**

The study involved 411 nurses working in 40 wards and clinics across both institutions.

**Methods:**

**:** A self-designed questionnaire was used to collect socio-demographic and work-related information, alongside standardized instruments: the Emotional Intelligence Questionnaire (INTE), the Perceived Stress Scale (PSS-10), and the Perceived Implicit Rationing of Nursing Care (PIRNCA). Descriptive statistics and both univariate and multivariate linear regression analyses were performed.

**Results:**

**:** The severity of nursing care rationing was significantly associated with stress level (B = 0.039, p < 0.001) and emotional intelligence (B = –0.006, p = 0.002). Nurses with secondary education and those working under civil law contracts reported significantly higher levels of care rationing. Additionally, care rationing was more prevalent among hospital nurses compared to those in other settings.

**Conclusions:**

**:** Low levels of emotional intelligence among Polish nurses coexist with high levels of stress and increased nursing care rationing. These findings indicate that emotional intelligence may serve as a protective factor that reduces the negative impact of stress on care provision.

**Trial Registration:**

**:** Not applicable.

**Social media abstract:**

**:** Rationing nursing care, in addition to the characteristics of the working environment, depends on the buffering role of emotional intelligence and the severity of stress experienced by nurses. #RationungNursingCare#QualityOfCare

**What is already known**

• Rationing of nursing care adversely affects the quality of care and contradicts the principles of holistic nursing practice.
• Stress in nurses can result from interactions with patients or from unfavorable working conditions, such as lack of autonomy, poor intergroup relations, insufficient support, and lack of adequate recognition.
• Emotional intelligence enables individuals to identify their own and others’ emotions, differentiate between them, and use this information to guide their reactions and behaviors.

Alt-text: Unlabelled box

**What this paper adds**

• Low levels of emotional intelligence among nurses are associated with higher stress levels and increased nursing care rationing.
• Nursing care rationing is influenced not only by working conditions but also by the buffering role of emotional intelligence in mitigating the effects of stress.

Alt-text: Unlabelled box

## 1 Background

The primary exponent of the quality of healthcare services, including nursing care, is ensuring patient safety. The work of nurses has a significant impact on achieving clinical outcomes, patient satisfaction and staff's own satisfaction with the care provided (Papastavrou et al. 2014). Nurses, struggling with limited resources and urgent responsibilities, often encounter difficulties, if not an inability, to fully implement all the requirements contained in individual nursing care plans. In addition, there may be risks in forcing nurses to shorten, delay or omit some elements of necessary patient care (Dhaini et al. 2019). In a staffing deficit situation, nurses are forced to ration care prioritising interventions based on clinical judgement. This practice may lead to a reduction or neglect of planned nursing care, which in turn increases the risk of negative outcomes for patients (Rochefort et al. 2016). As a result, the consequences of rationing nursing care are contrary to the principles of holistic nursing practice and adversely affect the quality of nursing care. According to Mandal et al. (2019) rationing of care also has a significant impact on the philosophical underpinnings of nursing practice.

Errors resulting from nurses' failure to act, as described by Harvey et al. (2018), Lake et al. (2016), Schubert et al. (2012) and Kalisch et al. (2012), among others, can lead to serious consequences such as prolonged hospitalisation, repeated hospitalisations, falls, infections, increased hospital mortality and reduced patient satisfaction. Nursing rationing, defined as limiting or failing to carry out necessary nursing activities due to lack of sufficient time, staff or skills, is now more openly recognised as a significant problem in healthcare (Schubert et al. 2008). In recent years, the concept of nursing rationing has been defined as the omission, delay or failure to implement necessary nursing interventions due to insufficient resources, such as staffing, time or equipment. It is a phenomenon that can lead to a deterioration in the quality of care and negative consequences for patients (Papierkowska, et al. 2024). The severity level of nursing rationing refers to the frequency and extent of limiting or omitting nursing activities due to insufficient resources, such as staff, time, or equipment, rather than the medical staff-to-patient ratio.

There are a number of reasons that lead to nursing rationing (Uchmanowicz et al. 2020). A 2022 systematic review of quantitative studies categorised the causes of nursing rationing into one of 3 groups, including:

1. organisational factors, including adequate staffing and resource availability, low rates of patients under nurse care, number of working hours, intensity of workload, high proportion of non-nursing tasks, overtime and unfavourable working conditions;
2. factors related to nurses such as absenteeism levels, job satisfaction or personal responsibility
3. patient-related factors, including clinical instability requiring intensive care, which can lead to nursing tasks being left unfulfilled (Chiappinotto et al. 2022).

To date, however, there has been no systematic review of qualitative research, despite the fact that this type of research has been used from the beginning to understand the causes of non-implemented nursing care, including through focus groups (Kalisch 2006).

The practice of professions based on interpersonal interactions is subject to natural exertion and emotions, which can lead to the experiencing of stress. Occupational stress is a psychological and physical reaction resulting from an inconsistency between the demands of the job and an individual's resources or abilities to cope with those demands. It is often associated with prolonged tension, fatigue, feelings of overwhelm and emotional burnout. This definition specifically emphasizes that occupational stress is a consequence of overloading the demands of the job relative to the resources available to nurses, which can negatively affect their mental and physical health ( Ghasemi Kooktapeh et al 2023; Quick et al. 2016).

In working with people, the ability to recognise and control emotions plays a key role. This aspect, called emotional intelligence by Salovey and Mayer (1990), describes the ability to identify one's own and others' emotions, differentiate between them, and use this information to guide one's own reactions and behaviours. Emotional intelligence is a measure of the ability to identify the nature of emotions and relationships, and to process and resolve issues based on them, and includes the perception of emotions, the identification of associated feelings, the understanding of information about emotions, and the control of emotions (Nikolau &Tsaousis 2002). Goleman (1998) emphasises that emotional intelligence is twice as important as technical skills and is more important than IQ for success in jobs at various levels. Weisinger (1998) suggests that it has a significant impact on career success and plays a key role in effective team leadership and group performance. It can be hypothesised that individuals demonstrating high levels of emotional intelligence will characterise their work environment as less stressful.

The study focuses on Polish nurses to take into account the peculiarities of the Polish health care system, which is characterized by limited resources and staffing shortages, affecting the rationalization of care.

Previous studies have shown that care rationing is associated with factors such as staff shortages, time pressure, and increased patient acuity. Research has mostly focused on organizational and workload-related determinants, while the role of psychological and emotional factors remains less explored. Although stress has been acknowledged as a contributor to clinical performance, few studies have directly examined its link to care rationing. Likewise, emotional intelligence (EI), which plays a critical role in coping with workplace demands, has rarely been investigated in this context. Therefore, this study aims to fill this gap by examining the potential relationships between EI, perceived stress, and care rationing among nurses.

The aim of this study was to examine factors that may influence the severity level of care rationing among Polish nurses.

The following dependent variables were established: overall rationing of care, Assistance with physical care, Monitoring-safety-support, Documentation-supervision, Communication, Implementation of prescribed treatment plan and independent variables: perceived stress, emotional intelligence, age, marital status, education, form of employment, place of work. Dependent variables, such as monitoring safety, communication, or the implementation of the treatment plan, are crucial for ensuring high-quality care, as widely discussed in the literature (Lake et al. 2016; Kalisch et al. 2012).

The following research hypotheses were formulated. Hypotheses H1–H2 were developed a priori based on the literature and the study design. One additional exploratory hypothesis (H3) was also considered.

H1: Higher levels of stress are associated with increased care rationing.

H2: Higher emotional intelligence is associated with decreased care rationing.

H3 (exploratory): The level of care rationing differs depending on the type of unit (surgical/specialist and primary care vs. internal medicine/long-term care).

## 2 Methods

### 2.1 Study design

This was a non-experimental, descriptive correlational study.

### 2.2 Sample and Sampling

A survey of nurses in north-western Poland was conducted in 2023.

#### 2.2.1 Inclusion and exclusion criteria

The sampling methodology employed was purposive sampling, targeting nurses working in healthcare facilities in north-western Poland. This approach ensured the inclusion of individuals with sufficient professional experience and relevant qualifications to provide meaningful data for the study's objectives. The method used was a diagnostic survey with the use of a paper survey questionnaire. Inclusion criteria for the study required participants to give informed and voluntary consent, hold a valid nursing licence, and be employed as a nurse in a healthcare facility for at least one year. The nurses surveyed had a valid license to practice: a nurse must be formally authorized to practice the profession, as evidenced by an appropriate document - a license to practice; they were employed in a health care facility providing care to patients within the scope of their employment in medical institutions; they had a minimum of one year's work experience: the survey included those who had worked in the profession for at least one year to ensure that the surveyed individuals had sufficient practice in the daily implementation of their professional duties. The exclusion criterion was lack of consent to participate in the study and incomplete survey responses.

The sample size for the study was calculated based on statistics from 2023 registers of professionally active nurses registered with the Szczecin Chamber of Nurses and Midwives. A confidence level of 95%, a maximum error of 5% and an estimated fraction size of 0.5 were used. Based on these parameters, the calculated with using the OpenEpi tool (OpenEPI), minimum participation required for the survey was 369 people (Szczecińska Izba Pielęgniarek i Położnych).

### 2.3 Data collection procedure

The study was conducted in Szczecin, Poland, in two university clinical hospitals: University Clinical Hospital No. 1 (comprising 28 wards and clinics) and University Clinical Hospital No. 2 (comprising 12 wards and clinics). Although email invitations to participate were initially sent to several healthcare facilities in the West Pomeranian region, only these two institutions provided formal administrative approval and took part in the study. Ultimately, two university clinical facilities in Szczecin that granted administrative approval were included in the study.

Questionnaires were distributed in paper-and-pencil format to nurses working in the approved departments. Participation was entirely voluntary and anonymous. Prior to completing the survey, all participants received written information outlining the purpose of the study, confidentiality protections, and their right to withdraw at any time without consequence. Informed consent was implied by the return of the completed questionnaire, in accordance with the procedure approved by the Bioethics Committee of the Pomeranian Medical University in Szczecin (ref. no. KB.006.52.2024, issued on March 27, 2024). Completed surveys were returned by participants into a locked collection box designated for this purpose.

In total, 500 questionnaires were returned. Of these, 89 were excluded due to incomplete data, resulting in 411 fully completed questionnaires included in the final analysis.

#### 2.3.1 Data collection

The researchers used a questionnaire developed by the authors to collect relevant socio-demographic and work environment information, including age, gender, marital status, place of residence, education, type and place of employment. In addition, standardised instruments such as the Emotional Intelligence Questionnaire (INTE), Perceived Stress Scale (PSS10) and Perceived Implicit Rationality of Nursing Care (PIRNCA) were used. Standardized survey refers to a pre-designed, validated tool that ensures consistency in data collection and allows comparison of results across different groups or settings.

The INTE questionnaire was developed to assess emotional intelligence, which is defined as the ability to recognise, understand and control one's own and others' emotions, and the ability to use emotions effectively in managing one's own and others' behaviour. Its design assesses the respondent's emotional intelligence and its two subscales: the ability to use emotions to support thinking and acting, and the ability to recognise emotions. For each of the sub-scales and for the total score, there are standard sten norms (separate for men and women) allowing the results of the questionnaire to be interpreted in terms of low, average and high scores. Stens 7-10 are assumed to be high scores, stens 5-6 are average scores and stens 1-4 are low scores. In the present study, adult norms were used (Jaworowska and Matczak, 2005; Jaworowska and Matczak, 2008).

• The INTE scale used in this study, developed by Jaworowska and Matczak (2005) and Jaworowska and Matczak (2008), is a widely used Polish measure of emotional intelligence. Internal consistency in the present sample was high, with a Cronbach’s alpha of 0.89.
• The tool has demonstrated good psychometric properties and construct validity in previous studies involving healthcare professionals and general populations in Poland.The PSS10 questionnaire provides an assessment of the degree of subjectively perceived stress, allowing an evaluation of the intensity of stress related to an individual's life situation over the past month (Cohen et al., 1983; Juczyński and Ogińska-Bulik, 2009). Sten norms exist for the PSS10. These allow the PSS10 to interpret scores. The most common assumption is that stens 5 and 6 represent average values, stens 7 to 10 represent high values and stens 1 to 4 represent low values. The PSS-10 scale, which measures the perceived level of stress, also demonstrated high internal consistency in this sample, with a Cronbach’s alpha of 0.86.
• The Polish adaptation of the tool was validated by Juczyński and Ogińska-Bulik (2009), confirming its reliability and construct validity in both clinical and non-clinical populations.
• The PIRNCA questionnaire consists of 31 statements describing nursing tasks that respondents were unable to perform due to insufficient resources (such as staffing or lack of time) during their last seven shifts. Each statement in the PIRNCA questionnaire is rated on a scale of 0-3, where 0 means 'never', 1 means 'rarely', 2 means 'sometimes' and 3 means 'often'. The overall care rationing score, which is the total score, is calculated as the average of all items. If none of the patients assigned to a nurse during these seven shifts required the corresponding activity, the respondent should mark it as 'not applicable'. The final PIRNCA score is the average score for all questions where a response was selected (questions marked 'not applicable' are excluded). Therefore, the total score varies between 0 and 3, with higher scores indicating more simplified nursing care. In addition, the questionnaire contains two follow-up questions. One concerns the nurses' assessment of patient care, rated from 1 to 10 on a Likert-type scale, where 1 means dangerously poor quality of care and 10 means very good quality of care. The second question concerns overall job satisfaction, also rated on a 10-point Likert-type scale, where 1 means "it's awful" and 10 means "I love it". According to the key, the PIRNCA questionnaire is divided into 5 sub-scales that indicate the degree of rationing of nursing care in each area: assistance with physical care, monitoring - safety - support, documentation - supervision, communication, and implementation of the prescribed treatment plan. The scores of the sub-scales are the mean scores of the questions within them (Zeleníková et al. 2020). The PIRNCA scale demonstrated excellent internal consistency in this study, with a Cronbach’s alpha of 0.92 for the total score. The subscale reliability coefficients were as follows: Physical care – 0.85, Monitoring-safety-support – 0.83, Documentation-supervision – 0.84, Communication – 0.82, and Implementation of prescribed treatment plan – 0.88. Regarding validity, the PIRNCA scale has shown good construct and criterion validity in previous international studies (Poghosyan et al., 2010), and the Polish version was culturally adapted and validated for use in healthcare settings (Uchmanowicz et al., 2021).

## 3 Data analysis

Quantitative variables were analysed by computing descriptive statistics, including mean, standard deviation, median, quartiles, and the range between minimum and maximum values. For qualitative variables, absolute frequencies and percentages of all possible values were calculated. Univariate and multivariate linear regression analyses were conducted to assess the effect of potential predictors on quantitative variables. Results are presented as regression parameters with 95% confidence intervals. The multicollinearity of multiple explanatory variables in the multivariate analysis was assessed using the Variance Inflation Factor (VIF), with VIF > 5 indicating multicollinearity and requiring removal of the relevant variables from the model. As the Subjects Per Variable (SPV) exceeded 10, no further variable selection was performed for multivariate analysis. A significance level of 0.05 was used, meaning that p-values below 0.05 were considered to indicate significant relationships. The analysis was performed using R software version 4.3.2 (R Core Team 2023).

### 3.1 Ethical considerations

The study protocol was approved by the Bioethics Committee of the Pomeranian Medical University in Szczecin on March 27, 2024 (ref. no. KB.006.52.2024). In addition, institutional permission to carry out the study was obtained from the participating healthcare facilities. The study design complied with the principles set out in the Declaration of Helsinki (World Medical Association Declaration of Helsinki: Ethical Principles for Medical Research Involving Human Subjects). Each participant received written information describing in detail the study objectives and procedures and gave written consent to participate in the study. Participants were also informed of their right to withdraw from the study at any time without giving a reason. The study used a printed questionnaire accompanied by a cover letter detailing its purpose, instructions for completion and expected duration.

## 4 Results

### 4.1 Characteristics of the study sample

In total, 500 questionnaires were distributed and collected; 89 were excluded due to missing data, leaving 411 complete questionnaires for the final analysis (response rate 82.2%).The analysis was conducted on this final dataset comprising 411 nurses working in Polish hospitals. The mean age in the study group was 39.86 years (SD=11.45), and the median was 42 years. The vast majority of respondents were women (93.93%), in formal relationships (56.45%) and living in large cities (40.39%). The largest proportion of the study group had a bachelor's degree (42.09%) and were employed in a highly specialised department (45.26%) under a contract of employment (66.67%) (Supplementary material Table 1).

**Table 1:** Level of emotional intelligence of respondents according to the INTE scale.

Result	INTE		
	INTE total score	Exploiting emotions	Recognition of emotions
Low scores	221 (53.77%)	224 (54.50%)	208 (50.61%)
Average results	111 (27.01%)	104 (25.30%)	132 (32.12%)
High scores	79 (19.22%)	83 (20.19%)	71 (17.27%)

### 4.2 Emotional intelligence, stress levels and rationing of nursing care among Polish nurses

The use of the INTE scale produced results indicating that 53.77% of the respondents had a low level of total emotional intelligence and 19.22% of the respondents had a high score in this area. Within the sub-scale of using emotions, 54.50% had a low score and 20.19% had a high score. On the emotion recognition scale, on the other hand, 50.61% of survey participants had a low score, while 17.27% had a high score (Table 1).

The PSS-10 scale allowed us to assess the severity of stress among the study group. High levels of stress were present in 62.04% of respondents, while low levels were present in 8.03% of study participants (Table 2).

**Table 2:** Level of stress intensity among respondents according to the PSS-10 scale.

PSS-10 - number of points	PSS10 Interpretation	n	%
0-13	Low stress levels	33	8.03%
14-19	Average stress level	123	29.93%
Over 19	High stress levels	255	62.04%

The mean score obtained by the respondents on the scale of general rationing of care was 1.05 (SD=0.69), rounding up to a score of 1, therefore indicating the infrequent occurrence of nursing rationing among the surveyed group of respondents. The area of nurses' activities most frequently affected by rationing was communication, with a mean of 1.26 (SD=0.81), while the problem was least frequent in the area of implementing the ordered treatment plan, with a mean of 0.75 (SD=0.75) (Table 3).

**Table 3:** Analysis of severity of care rationing with sub-scales according to PIRNCA.

Parameter	N	Data gaps *	Range of values	Average	SD	Median	Min	Max	Q1	Q3
Rationing of care	409	2	0-3	1.05	0.69	1.00	0	3	0.53	1.45
Assistance in physical care	394	17	0-3	1.14	0.79	1.12	0	3	0.50	1.75
Monitoring-Security-Support	404	7	0-3	1.09	0.75	1.00	0	3	0.50	1.62
Documentation-Supervision	398	13	0-3	1.04	0.81	1.00	0	3	0.33	1.65
Communication	393	18	0-3	1.26	0.81	1.25	0	3	0.67	2.00
Implementation of the recommended treatment plan	393	18	0-3	0.75	0.75	0.60	0	3	0.00	1,00

* or "Not applicable" answers alone

Within the PIRNCA scale, respondents provided a response regarding their subjective assessment of the quality of patient care, for which the mean was 6.94 points (SD=1.95), and their assessment of job satisfaction, for which the mean was 6.11 points (SD=2.21); (Supplementary material Table 2).

Exploring factors influencing the level of rationing of nursing care by Polish nurses

Univariate linear regression models were developed to assess the influence of individual factors on the overall level of care rationing and care rationing in each of the 5 areas forming the PIRNCA subscales.

### 4.3 Findings related to Hypothesis 1 (Stress and Care Rationing)

These showed that stress level significantly influenced care rationing (p<0.001); on average, each additional stress point increased care rationing by 0.039 points. In contrast, each additional point of general emotional intelligence, on average, decreased the level of care rationing by 0.006 - a relationship that applied to all areas of care, except communication. Each additional point within emotion literacy reduced the level of care rationing by 0.011 points on average.

### 4.4 Findings related to Hypothesis 2 (Emotional Intelligence and Care Rationing)

The univariate models also showed that the place of work influenced the level of care rationing in all areas except implementation of prescribed treatment plan. Working on a surgical ward reduced the level of care rationing by an average of 0.287 points compared to working on an internal medicine or long-term care ward. In contrast, working in a highly specialized ward reduced the level of care rationing by an average of 0.306 points relative to working in an internal medicine or long-term care ward, and working in a primary care clinic reduced the level of care rationing by an average of 0.58 points relative to working in an internal medicine or long-term care ward (Table 4).

**Table 4:** One-factor model of the influence of stress, emotional intelligence and socio-demographic factors on the level of care rationing.

Feature		Holistic rationing of care		Assistance with physical care		Monitoring-safety-support		Documentation-supervision		Communication		Implementation of prescribed treatment plan	
		Par.	p	Par.	p	Par.	p	Par.	p	Par.	p	Par.	p
PSS-10		0.039	<0.001*	0.038	<0.001*	0.029	<0.001*	0.033	<0.001*	0.031	<0.001*	0.02	0.001*
INTE total score		-0.006	0.002*	-0.005	0.023*	-0.005	0.022*	-0.008	<0.001*	-0.004	0.055	-0.006	0.003*
Exploiting emotions		-0.011	0.001*	-0.01	0.01*	-0.009	0.012*	-0.016	<0.001*	-0.009	0.024*	-0.012	0.001*
Recognition of emotions		-0.01	0.05	-0.007	0.225	-0.008	0.157	-0.018	0.003*	-0.006	0.292	-0.011	0.05
Age [years]		0.004	0.154	0.006	0.077	0.002	0.493	0.005	0.173	0.01	0.004*	0.001	0.697
Marital status	Formal relationship	ref.		ref.		ref.		ref.		ref.		ref.	
	Informal relationship	0.043	0.603	0.005	0.962	0.074	0.422	0.028	0.78	-0.067	0.502	0.162	0.081
	Free	0.041	0.645	0.041	0.696	0.091	0.354	0.006	0.958	-0.101	0.347	0.042	0.666
Education	Medium	ref.		ref.		ref.		ref.		ref.		ref.	
	Higher 1st degree	0.081	0.33	0.078	0.428	0.115	0.213	0.023	0.814	-0.1	0.327	0.19	0.042*
	Higher 2nd or 3rd degree	0.033	0.712	0.011	0.917	0.044	0.657	0.078	0.47	-0.084	0.444	0.162	0.107
Form of employment	Employment contract	ref.		ref.		ref.		ref.		ref.		ref.	
	Civil law contract or commission	-0.05	0.485	-0.038	0.652	-0.062	0.439	-0.049	0.566	-0.004	0.959	-0.016	0.841
Place of employment	Internal medicine or long-term care unit	ref.		ref.		ref.		ref.		ref.		ref.	
	Treatment department	-0.287	0.003*	-0.323	0.005*	-0.294	0.006*	-0.283	0.015*	-0.342	0.004*	-0.153	0.162
	High-speciality department	-0.306	0.001*	-0.311	0.004*	-0.35	0.001*	-0.213	0.053	-0.299	0.007*	-0.178	0.085
	Primary care clinic	-0.58	0.002*	-0.768	<0.001*	-0.629	0.002*	-0.522	0.018*	-0.671	0.003*	-0.379	0.067

* statistically significant relationship (p<0.05), Par - parameter

A multivariate linear regression model showed that the level of nursing rationing was influenced by a number of factors, related to stress level, emotional intelligence and the characteristics of the study group and its workplace. An increase in stress level by each additional point raised the level of care rationing by an average of 0.004 points, while each additional point of general emotional intelligence lowered the level of care rationing by an average of 0.035 points. In all PIRNCA sub-scales analysed, the values indicated statistically significant relationships between the study variables.

In addition to these two pre-specified hypotheses, we conducted exploratory analyses of socio-demographic variables, as described below.

Among the socio-demographic factors, it was shown that: each additional year of age increased the level of care rationing by an average of 0.012 points. Age had a significant impact on care rationing in each of the areas analysed. Other socio-demographic factors such as marital status and educational level influenced the overall level of rationing and some of the analysed areas of nursing care. Those who declared that they were in an informal relationship showed an average of 0.213 points higher level of overall rationing of care compared to those who were married. First degree education resulted in an average 0.184 points higher level of care rationing compared to secondary education. The place of work was also important for the level of care rationing: working in a surgical ward reduced the overall level of rationing by an average of 0.271 points relative to working in an internal medicine or long-term care ward, while working in a highly specialised ward reduced the level by an average of 0.265 points relative to working in an internal medicine or long-term care ward, and working in a primary care clinic reduced it by an average of 0.557 points (because the regression parameter is -0.557) relative to working in an internal medicine or long-term care ward (Table 5).

**Table 5:** Multivariate model of the influence of stress, emotional intelligence and socio-demographic factors on the level of care rationing.

Feature		Rationing of care		Assistance with physical care		Monitoring-safety-support		Documentation-supervision		Communication		Implementation of prescribed treatment plan	
		Par.	p	Par.	p	Par.	p	Par.	p	Par.	p	Par.	p
PSS-10		-0.004	0.034*	0.04	<0.001*	0.03	<0.001*	0.036	<0.001*	0.033	<0.001*	0.023	<0.001*
INTE total score		0.035	<0.001*	-0.007	0.001*	-0.006	0.003*	-0.01	<0.001*	-0.006	0.006*	-0.007	<0.001*
Age [years]		0.012	0.002*	0.013	0.002*	0.01	0.021*	0.012	0.011*	0.014	0.003*	0.013	0.004*
Marital status	Formal relationship	ref.		ref.		ref.		ref.		ref.		ref.	
	Informal relationship	0.213	0.03*	0.219	0.051	0.225	0.038*	0.21	0.071	0.183	0.124	0.316	0.005*
	Free	0.08	0.367	0.072	0.479	0.124	0.205	0.025	0.809	-0.069	0.518	0.095	0.341
Education	Medium	ref.		ref.		ref.		ref.		ref.		ref.	
	Higher 1st degree	0.184	0.041*	0.243	0.02*	0.225	0.024*	0.161	0.134	0.079	0.477	0.299	0.004*
	Higher 2nd or 3rd degree	0.153	0.095	0.187	0.077	0.16	0.118	0.221	0.044*	0.097	0.387	0.293	0.006*
Form of employment	Employment contract	ref.		ref.		ref.		ref.		ref.		ref.	
	Civil law contract or commission	-0.008	0.909	0.037	0.653	0	0.998	0.026	0.76	0.056	0.52	0.028	0.724
Place of employment	Internal medicine or long-term care unit	ref.		ref.		ref.		ref.		ref.		ref.	
	Treatment department	-0.271	0.004*	-0.32	0.003*	-0.289	0.006*	-0.268	0.016*	-0.342	0.003*	-0.159	0.133
	High-speciality department	-0.265	0.003*	-0.281	0.005*	-0.334	0.001*	-0.184	0.078	-0.274	0.01*	-0.169	0.09
	Primary care clinic	-0.557	0.002*	-0.724	<0.001*	-0.617	0.002*	-0.537	0.012*	-0.618	0.006*	-0.444	0.028*

* statistically significant relationship (p<0.05), Par - parameter

Across models, the association with care rationing was stronger for perceived stress than for emotional intelligence. For example, in the univariate analysis (Table 4) the coefficient for stress was β=0.039 compared to β=−0.006 for emotional intelligence. In the multivariate analysis (Table 5), the effects of stress (β≈0.02–0.04) also consistently exceeded those of emotional intelligence (β≈−0.006 to −0.010) across PIRNCA subscales.

## 5 Discussion

Nursing rationing, defined as delaying, interrupting or completely omitting certain aspects of care due to limited resources, is a major challenge in the context of providing quality health services. The purpose of the study was to examine the effect of stress level and emotional intelligence on care rationing.

### 5.1 Interpretation of Hypothesis 1: Stress and Care Rationing

It was hypothesized that there is a positive correlation between stress level and care rationing, which was confirmed by the results of our study. It has also been suggested that emotional intelligence negatively correlates with care rationing. Our findings suggest that nurses with higher levels of emotional intelligence tended to report lower levels of care rationing. This association may indicate a meaningful relationship worth exploring further in future research.

In a study of a sample of 1,353 nurses from the Czech Republic, Slovakia, Croatia and Poland, five most frequently rationed activities were identified: emotional or psychological support, timely response to patient or family request or need, important discharge conversations, patient and family education and appropriate supervision of delegated tasks (R Core Team 2023). These results are consistent with the findings of Jones et al. (2015), who also demonstrated that certain aspects of nursing care are regularly overlooked or delayed. Despite reporting a variety of uncompleted nursing activities, the mean results suggest that the frequency of rationing nursing care ranges from a response of 'never' to 'rarely'. In Poland, an assessment of the severity of nursing rationing has also been addressed, with similar results (Koltuniuk et al. 2021; Jarosz et al. 2022; Witczak et al. 2022; Piotrowska et al. 2022; Radosz-Knawa et al. 2022; Uchmanowicz et al. 2021; Marczak & Milecka 2024) showing a relatively low declarative frequency of nursing rationing. These results are consistent with those obtained in the present study: on average, nurses declared a rare occurrence of care rationing situations. Despite the low level of rationing, it is interesting to note that activities related to communication were the most frequently rationed, while instrumental implementation of the ordered treatment plan was the least frequently rationed, which may reflect nurses' beliefs regarding the prioritisation of particular activities. According to Jones et al. (2021), nurses tend to overlook those nursing activities that: in their judgement are unlikely to have a direct or immediate impact on patients' health; are time-consuming and cannot be shortened; the time required to complete them is unknown or difficult to estimate; and are less likely to be checked on the ward. These findings apply fully to the results obtained: communication activities meet these Jones criteria, whereas the completion of medical orders definitely does not. Meanwhile, suboptimal information exchange can have dire consequences for patient safety and life. Confirmation can be found in the risk management literature, where communication errors are considered the main cause (70%) of adverse events (Guttman et al. 2021). The aforementioned research on nursing rationalisation therefore highlights the relevance of the problem and the need to take action to optimise resources in the healthcare system to ensure that all aspects of nursing care are fully implemented.

Research on the relationship between the level of emotional intelligence and the severity of care rationing indicates a significant correlation between these variables. Goleman (2020) defines emotional intelligence as the ability to recognise, understand and manage one's own emotions and those of others. A study by Codier and Codier (2015) found that nurses with higher levels of emotional intelligence are characterised by greater effectiveness in managing emotions in stressful clinical situations and a greater ability to provide complete care to patients, even in the face of limited resources, through better time management and task prioritisation. Chrzan-Rodak & Slusarska (2019) suggest that higher levels of emotional intelligence may act as a protective measure against professional burnout. They pointed out that emotional intelligence and social competence are key in interpersonal interactions and can contribute to better quality nursing care.

It is worth noting that the results of studies on the level of emotional intelligence among nurses may vary between countries. For example, a recent study of nurses working in the Portuguese national health service found moderate levels of emotional intelligence, with variation depending on age and professional experience (Neves et al., 2024). These differences may be influenced by national healthcare systems, work environments, and educational frameworks shaping emotional competency development in the nursing profession.

In contrast, research into the impact of stress on levels of nursing rationing indicates a significant relationship between these variables. Nurses' occupational stress, as a result of high workloads, understaffing and the emotional demands of caring for patients, can lead to increased levels of care rationing. The results of our own research indicate that high levels of stress significantly increase the rationing of nursing care. Analysis of the effect size showed that an increase in stress level by each additional point on the PSS-10 scale raised the level of care rationing by 0.004 points, which may seem like a small effect. However, in clinical practice, under conditions of chronic stress and staff shortages, even such differences can lead to a significant reduction in the quality of care provided. In the long term, the cumulation of such effects can cause significant consequences for patient safety and nurses' job satisfaction. These findings suggest that stress reduction programs and support mechanisms for staff could be valuable, and their impact warrants further investigation.

Studies conducted in recent years indicate persistently high levels of occupational stress among nurses. A study by Wilczek- Rużyczka et al. (2019) found that 95.6% of nurses surveyed consider their profession stressful, and 56.1% experience daily occupational stress. The main sources of stress are excessive responsibility for patients' lives and time pressure. Another study highlights that nurses and midwives often experience stress related to their broad job responsibilities, the pressure to be reliable, and expectations from patients and their families (Kaźmierczak et al. 2019).

A study by Tomaszewska et al. found that job burnout and low job satisfaction are significantly associated with nurses' rationing of care in intensive care units, which can lead to a decrease in the quality of care provided (Tomaszewska et al. 2024). Another study by Babapour et al. (2022) found that high levels of occupational stress in nurses negatively affect their quality of life and caregiving behavior, which can result in poorer health outcomes for patients.

A study conducted in earlier years by Schaufeli and Bakker (2004) found that high levels of occupational stress correlate with lower quality of care provided and higher levels of rationing. Specifically, nurses experiencing high levels of stress are more likely to reduce the amount of time they spend with patients and limit the performance of nursing procedures, leading to poorer patient health outcomes. Similar results were obtained by McHugh et al. (2011), who found that occupational stress influences resource allocation decisions and task prioritisation in nursing care. In high-stress settings, nurses are forced to make difficult choices that often result in rationalisation of care, particularly in the context of tasks considered less urgent or less critical.

### 5.2 Interpretation of Hypothesis 2: Emotional Intelligence and Care Rationing

In our own study, higher levels of emotional intelligence correlated with lower levels of care rationing. The observed association between emotional intelligence and care rationing suggests that emotional intelligence competencies could be an important focus for further investigation in nursing research.(a reduction of 0.035 points in the multivariate model).

A study by Almeneessier & Azer (2023) found an inverse relationship between levels of emotional intelligence and job burnout among academics and clinicians, suggesting that higher emotional intelligence may protect against job stress. Similarly, a study by Chen et al. (2024) confirmed that emotional intelligence is negatively correlated with job burnout, suggesting that people with higher emotional intelligence are better able to cope with stress at work.

Furthermore, emotional intelligence, understood as the ability to recognise, express and control emotions, can have a significant impact on perceived occupational stress and the consequences of experienced stress. Gardner and Stough (2003) showed a negative relationship between the level of emotional intelligence and perceived occupational stress. Slaski and Cartwright (2002) also found that managers with high emotional intelligence experienced less subjective stress and had better physical and psychological well-being. A study of police officers by Bar-On et al. (2000) indicated that those scoring significantly higher on emotional intelligence were less susceptible to and better able to cope with the stress experienced. Similar results were obtained by Duran and Extremera (2004), who, in their study involving professionals working in institutions for people with intellectual disabilities, revealed a significant relationship between emotional intelligence and the burnout syndrome, particularly with the sense of personal achievement.

The relationship between emotional intelligence, perceived stress and care rationing is an important area of research with key implications for the quality of healthcare. In the context of care rationing, high emotional intelligence may reduce the tendency to overlook or delay certain aspects of care. Nurses with higher levels of emotional intelligence appear to be better equipped to manage their emotions and stress, which may lead to more effective performance of duties and minimisation of care rationing.

In Poland, the health care system is mainly based on public health care, funded by the National Health Fund. Nurses are employed in both public and private facilities, but most work in the public sector. The characteristics of the system, including limited resources, understaffing and high professional demands, may affect the level of occupational stress and emotional intelligence among Polish nurses.

In conclusion, the current research confirms a significant relationship between occupational stress and emotional intelligence among nurses. International comparisons suggest that differences in the level of emotional intelligence may be related to the peculiarities of health care systems and working conditions in each country.

The finding that rationing of care was higher in internal medicine and long-term care units compared with surgical or specialty wards is not unexpected. However, several practical strategies could help mitigate this problem. These include: (1) reducing non-nursing tasks through additional support staff; (2) introducing protected time for nurse–patient and nurse–family communication, as communication was the domain most often rationed in our study; (3) implementing structured handover and bedside huddles to prioritize care; (4) adjusting staffing to patient case-mix; (5) using electronic health record templates and checklists to streamline documentation and education; (6) establishing a float pool to cover workload peaks; and (7) offering brief, regular training to enhance emotional and communication skills. Future research should evaluate the effectiveness and feasibility of these strategies in high-risk units.

### 5.3 Implications

Our own research shows that high levels of stress significantly influence care rationing. Our findings suggest that interventions aimed at stress reduction—such as mindfulness or psychological support programs—could be explored as potentially beneficial for improving staff well-being and care quality. However, further studies are needed to assess their effectiveness in reducing care rationing. In addition, future research might examine whether systematic monitoring of stress levels and care quality could help identify areas of concern and inform support efforts. Our findings suggest that emotional intelligence competencies may play an important role in nursing practice and could be a valuable subject of further research. Given the observed association between emotional intelligence and care rationing, future research could investigate whether training programs focused on emotional competencies might support nurses in managing workload-related stress and prioritizing patient needs more effectively. Training aimed at enhancing emotional intelligence may help nurses with teamwork, time and task management. However, its actual impact on care rationing should be further investigated. Our results suggest that future research might examine whether incorporating emotional skills development into nursing education has the potential to positively influence care quality. Potential avenues for such training could include workshops, clinical simulations, or communication-focused sessions, though their effectiveness should be evaluated in future studies. Given the frequent rationing of communication-related tasks, future studies could explore how workplace communication structures and practices influence care delivery and prioritization.

Older age and lower levels of education were associated with higher levels of care rationing. Future studies could examine whether training programs tailored to varying levels of proficiency and experience are associated with reduced care rationing.

A work environment that fosters mutual support, autonomy, and appreciation may contribute to lower stress levels and could potentially reduce care rationing – an area that warrants further study.

These associations point to several areas—such as communication training, emotional competency, and team dynamics—that may benefit from targeted intervention, pending further study.

In light of the stronger association of stress (compared with emotional intelligence) with care rationing (Table 4, Table 5), the primary direction for interventions should be strategies aimed at reducing stress and workload, complemented by training in emotional and communication skills

### 5.4 Exploratory Findings: Socio-demographic and Workplace Factors

Although not included in the primary research hypotheses, exploratory analyses revealed that several socio-demographic and workplace variables were significantly associated with the level of nursing care rationing. Specifically, higher levels of rationing were reported among nurses with secondary education, those employed under civil law contracts, and those working in hospital settings. These findings suggest that institutional and employment-related factors may contribute to nurses’ ability to provide complete care. While these results were not hypothesized a priori and should be interpreted with caution, they indicate potentially important areas for future research and workforce policy development.

### 5.5 Limitations

This study assessed self-reported, perceived care rationing, which may differ from objectively observed care behaviors. Future research should consider observational or record-based designs to validate perceived versus actual care omissions.

The survey included only nurses employed in hospitals in northwestern Poland. The results may not reflect the specifics of nurses' work in other regions of Poland or in other countries. To get a more comprehensive picture of the phenomenon, it is necessary to include healthcare facilities in other European countries, which could provide comparative data.

The study did not take into account all potential factors related to nurses, patients and the organization of nursing work that may influence care rationing.

In addition, a self-report survey method was used, which could have led to nurses giving socially desirable answers instead of fully honest ones.

The survey focused on specific variables, such as emotional intelligence and stress levels. Other potential variables that may play a role in rationing nursing care were not considered.

In addition, the study was cross-sectional, making it impossible to track changes in nursing rationing over the long term and to assess the effects of implemented interventions.

The vast majority of respondents in the survey were women, reflecting the demographic structure of the nursing profession in Poland. However, the limited representation of men in the sample may affect the generalization of the results. The diversity of professional and demographic experiences was limited by the composition of the study group, suggesting the need for further research with greater inclusion of men.

It should also be noted that one of the limitations of the study is the lack of analysis of the characteristics of the 18% of respondents who did not complete the survey. Potential differences between the group of respondents and the excluded group, may affect the results of the survey, suggesting the need for further analysis in this regard.

Highlighting these limitations is crucial to understanding the study's findings and may provide a starting point for further, more comprehensive analyses.

## 6 Conclusions

1. Rationing of nursing care occurs sporadically, but is most frequently related to communication, suggesting that this area may warrant particular attention in future research and practice.
2. Our findings suggest that stress may play a role in care rationing, indicating that stress-reduction efforts could be a worthwhile area for further investigation.
3. Similarly, the observed association between higher emotional intelligence and lower care rationing suggests that emotional intelligence may be a promising area for further research, including potential interventions.
4. Our results indicate that working conditions and nurse characteristics may be associated with care rationing levels. These patterns highlight the value of further research into tailoring support strategies to nurses’ diverse contexts
5. These findings point to areas—such as soft skills and environmental factors—that may merit attention in future research aiming to support nurses and enhance care quality

## Funding sources

This research received no external funding.

## CRediT authorship contribution statement

**Daria Schneider-Matyka:** Writing – review & editing, Validation, Resources, Data curation, Conceptualization. **Kamila Rachubińska:** Visualization, Software, Resources, Project administration, Conceptualization. **Anna Maria Cybulska:** Software, Resources, Project administration, Formal analysis, Conceptualization. **Mariusz Panczyk:** Methodology. **Przemysław Ustianowski:** Validation, Software, Resources. **Ireneusz Walaszek:** Software, Resources, Data curation. **Dorota Ćwiek:** Software, Resources, Data curation. **Elżbieta Grochans:** Writing – review & editing, Supervision, Funding acquisition, Conceptualization. **Małgorzata Szkup:** Writing – original draft, Conceptualization.

## Declaration of competing interest

The authors declare that the research was conducted in the absence of any commercial or financial relationships that could be construed as a potential conflict of interest.

## Appendix Supplementary materials

Image, application 1
