# Supplementary material for: Factors associated with nursing care rationing in Poland: A cross-sectional observational study
Source: Int J Nurs Stud Adv. 2025 Aug 27;9:100413. doi: 10.1016/j.ijnsa.2025.100413 (PMC12446665; doi:10.1016/j.ijnsa.2025.100413)
Supplement: Supplementary file 1 [file mmc1.docx]

**Supplementary material**

Table 1. Characteristics of the study sample

| Parameter | | Total (N=411) |
| --- | --- | --- |
| Age [years] | Mean (SD) | 39.86 (11.45) |
|  | Median (quartiles) | 42 (29-49) |
|  | Scope | 21-67 |
| Gender | Woman | 386 (93.92%) |
|  | Male | 25 (6.08%) |
| Marital status | Formal relationship | 232 (56.45%) |
|  | Informal relationship | 98 (23.84%) |
|  | Free | 81 (19.71%) |
| Place of residence | Village | 73 (17.76%) |
|  | City up to 10.000 inhabitants | 51 (12.41%) |
|  | City of 10.000-100.000 inhabitants | 121 (29.44%) |
|  | City of over 100.000 inhabitants | 166 (40.39%) |
| Education | Medium | 112 (27.25%) |
|  | Higher 1st degree | 173 (42.09%) |
|  | Higher 2nd or 3rd degree | 126 (30.66%) |
| Type of employment | Employment contract | 274 (66.67%) |
|  | Civil law contract or commission | 137 (33.33%) |
| Place of employment | Internal medicine or long-term care unit | 76 (18.49%) |
|  | Treatment department | 132 (32.12%) |
|  | High-speciality department | 186 (45.26%) |
|  | Primary care clinic | 17 (4.14%) |

Table 2. Nursing assessment of quality of patient care and job satisfaction

| PIRNCA | N | Data gaps | Range of values | Average | SD | Median | Min | Max | Q1 | Q3 |
| --- | --- | --- | --- | --- | --- | --- | --- | --- | --- | --- |
| Assessing the quality of patient care | 411 | 0 | 0-10 | 6,94 | 1,95 | 7 | 2 | 10 | 6 | 8 |
| Job satisfaction assessment | 411 | 0 | 0-10 | 6,11 | 2,21 | 6 | 0 | 10 | 5 | 8 |

SD - standard deviation, Q1 - lower quartile, Q3 - upper quartile
